# Sulphuric Acid for Opening Root Canals

**Published:** 1894-02

**Authors:** J. R. Callahan

**Affiliations:** Cincinnati, O.


### COMMUNICATIONS.

### Sulphuric Acid for Opening Root-Canals.

BY J. R. CALLAHAN, D.D.S., CINCINNATI, O.

Read before the Ohio State Dental Society, December, 1893.

The opening of "Root Canals" has been the subject of much discussion and many wild assertions. We have often heard of dentists who claim to be able to open to the apex, the root canal or canals in any tooth.

I wish to say that no such claim will be made for the method that I desire to bring before you to-day. For the opening or enlarging of all straight and unobstructed canals, I prefer and use the "Gates Glidden Drill," after the manner described by Dr. Geo. Evans, in his work on "Artificial Crown and Bridge-Work," Third Edition, (page 21), but, valuable as this instrument may be, we see almost every day in practice, canals that cannot be opened properly by this or any other drill. It is seldom that we see canals in buccal roots of superior molars, or in roots of lower molars, in which a drill can be used; many times in bicuspids and inferior incisors the roots are so flat and thin that drilling is dangerous; yet all these canals may be in such condition that we are compelled to open them for treatment and filling. There are canals that are constricted just at the chamber, sometimes so much so that they can scarcely be found, yet the canal in the root is large and should be opened. There are the canals in curved roots, and canals obstructed by osseous growths, that if not properly opened would most likely cause trouble. It is with this difficult class of root-canals that I wish to deal at this time.

It has been about four years since I began to open this difficult class of canals by using a 20 to 50 per cent. aqueous solution of sulphuric acid and the Donaldson Root-Canal Cleanser. To illustrate the method, let us suppose we have a superior molar tooth, from which the pulp tissue has been removed, the palatine root being large, can be prepared by any method you may choose; but let us say, the canals in the buccal roots cannot be found, we would then place a pledget of cotton, saturated with the acid solution in that portion of the cavity near the buccal roots and seal it in the tooth for twenty-four or forty-eight hours; then upon removal of the stopping, wash out the cavity with a dash of water from the syringe, upon drying the cavity you will find it white and clean, with two dark spots in the vicinity of the buccal roots, showing where the canals can be found.

Now we try to enter the canal with the nerve bristle, we find no opening; to make sure we are not being deceived by a constriction, we take a bud drill and follow these stains a short distance. If we find no opening or a very minute opening too small for the bristle, we will feel justified in saying they need no further treatment. But, if with an exploring instrument a canal is found, we will carry the acid to the canal by dipping the instrument in the solution, or by means of the pliers, or better still with the latest pattern of the Dunn Syringe place a drop of the agent in the chamber, (the Dunn Syringe referred to is made of glass and rubber with a platinum or gold point), and with a No. 5 Donaldson Canal Cleanser pump the acid into the canal; the acid will soften the walls of the canal sufficient to allow the broach to open its way into the root; the acid will also thoroughly sterilize the canal and everything in it. No germ or spore can live in the presence of $H_2SO_4$ in the strength mentioned. The broach may scarcely enter the canal at first, but if you are persistent it will be but a few minutes till the instrument will go quite a distance into the canal until you reach the end of the root, where a much stronger resistance will be met with. The thickened cementum at this point, seeming to offer a greater resistance to the agents used. The canal can then be enlarged by using larger broaches, or if the root-canal is straight the Gates Glidden Drill will follow the canal just made. It is more than likely that the apical foramen has not yet been opened; this can be accomplished, if desired, by drilling or by placing a small thread of cotton saturated with the acid solution in the end of the root and leaving it there over night, then using the broach and acid at the next sitting; after one or two trials you can readily see how crooked or obstructed canals may be opened in a few minutes, and the canal will be in condition for immediate root filling. It must be borne in mind that the rubber dam should always be in place before the operation is begun; the adjoining teeth may be protected by placing the dam only on the tooth being operated on.

I confess that at first sight, the application of so strong a solution as 50 per cent. looks to be rather heroic; (good results may be obtained by the use of weaker solutions, but my desire to present a ten-minute paper prohibits the mentioning of many details), but four years constant use has proven to me that there is little or no danger of injuring the tooth or the surrounding tissue, if the operation be controlled by any sort of common sense. We do not hesitate to use arsenic, or nux vomica, aconite, argenti nitras, cocaine, and scores of other poisonous drugs. We can have the action of the acid under perfect control. I always keep a saturated solution of bicarbonate of soda on the case so that I may stop the action of the acid at any moment. In but few cases is it probable that the acid will go through the apical foramen in quantities or strength sufficient to have any corrosive effect, for the reason that neutralizing agents in the dentine will have materially weakened the acid before it can pass through the extremely small opening at the apex of the root; if there be an abscess present the foramen is likely to be larger and the condition of the tissues about the apex of the root will be materially benefited by the presence of the acid even if in the full strength of the solution. In my mind there could be no better agent for the breaking down of the diseased tissues and the positive destruction of all germ life.

A case in practice will probably illustrate the point I wish to develop: A lady about twenty-five or thirty years of age who had been under surgical treatment for a large fistula at the symphysis of the chin, came to me at the request of the surgeon for examination and treatment, if I thought the case demanded it; by the aid of the electric light I was enabled to locate the trouble in the two inferior central incisors; the pulp-chambers in both teeth were opened, the canal in each tooth was so small that they were practically closed; a drop of the acid solution was placed in the pulp-chamber, and with a No. 5 Donaldson Root-Canal Cleanser was pumped into the canal, in a few minutes the instrument found its way through the root, the canals were then enlarged by using larger broaches, thereby establishing direct communication from the pulp-chamber through the seat of the abscess and through the roots into the fistula, and the acid made its escape through the opening at the symphysis. On the second day the case was given the usual antiseptic dressing; on the fourth day the roots and fistula were thoroughly filled with chlora-percha. The case was kept under observation for a few days, no signs of inflammation appearing the case was dismissed as cured. This is one of a number of cases successfully treated in this manner. In this case I do not believe the roots could have been opened in a reasonable time by any other method, and I also believe that the acid solution was the best remedial agent that could have been applied at that stage of the treatment. The acid at first attacks the tooth substance vigorously, breaking up the lime salts, and corroding or changing the form of the organic substance and forming a new compound, thereby establishing a barrier to the further progress of the acid.

Prof. J. S. Cassidy, in his valuable text-book, "Dental Chemistry and Materia Medica" (page 77), says: "The acid attacks the earthy portion forming insoluble calcium sulphate, ($CaSO_4$), and at the same time dehydrating the animal or gelatinous portion, which is mainly made up of carbon, hydrogen and oxygen; these two latter elements are withdrawn as already alluded to, leaving the indestructible carbon as a residue, to be incorporated with the insoluble sulphate, producing thus, a protecting covering to the unaffected parts beneath, against further inroads both of the causing agent and other solvents."

I have here several specimens which I hope you will examine carefully. Large cavities were cut in sound teeth; the cavity in No. 1 was kept full of a 50 per cent. (by volume), solution of sulphuric acid for ten minutes. The cavity in No. 2 was kept full of same solution for two hours. The cavity in No. 3 was kept full of same solution for twenty-four hours. The cavity in No. 4 was subjected to no other treatment than to be washed with alcohol. The cavity in No. 5 was filled with same solution on December 1, at 1 P. M., and kept full till the morning of December 5, when I placed a pledget of cotton saturated with the solution in the cavity, and sealed it in the tooth with wax. This was done that I might bring the tooth to you, that you may open and examine for yourselves. In No. 5 the root canals were opened as described, by the use of the acid and broaches, and the ends of the roots were then ground off to show the action of the acid on the walls of the root-canals near the apex. No. 6 received no treatment further than to have the ends of the roots ground off the same as No. 5, in order to show the normal appearance of the root-canal near the apex. I think you will be unable to discover any material difference in the appearance of the dentine in Nos. 1, 2 and 3, which I think is proof that the action of sulphuric acid on tooth substance is self-limited, for we find in this experiment that the acid penetrated the tooth substance, practically, no farther in twenty-four hours than it did in ten minutes. If with an excavator the almost invisible layer of dissolved dentine be removed, a perfectly hard, smooth surface will be found. About the root-canals, specimen No. 5, you will see what appears to be a small zone of decalcified dentine. A glance at No. 6 will show the same appearance. As to the action of the acid solution on the bone surrounding the roots of the teeth, I quote Mr. George Pollock, F.R.C.S., Surgeon to St. George's Hospital, who says: "Dilute sulphuric acid does not affect the living bone, acting chemically on diseased bone alone," and gives the following experiments: "Portions of dead diseased, and healthy bone were selected and subjected to the action of sulphuric acid, viz.:

| | | |
|---|---|---|
| No. 1. | Dead bone, | 10 grains. |
| " 2. | Diseased bone, | " " |
| " 3. | Healthy bone (middle age), | " " |
| " 4. | Healthy bone (old age), | " " |

" Exposed to the action of a mixture of sulphuric acid and water, one part in four, for three days, at a temperature of 100 degrees, the following were the results:

" No. 1. Dead bone : Phosphate of lime 2 grs. Carbonate of lime 3.30 grs. dissolved in the mixture.

" No. 2. Diseased bone: Phosphate of lime 2 grs. Carbonate of lime 1.3 grs. dissolved in the mixture.

" Nos. 3 and 4. In both specimens of healthy bone. *No action took place.*"

Prof. Garretson says, in the treatment of " Caries of the Maxillæ," he has used the " officinal ordinary sulphuric acid." (Garretson's Oral Surgery, Third Edition, pages 705 and 706.)

On the soft tissues the solution will have a corrosive and astringent effect, or in other words will break down or destroy the diseased tissue, leaving a fresh, clear field for nature to take care of herself, with the assistance of milder antiseptic treatment.

